# Effects analysis of reward functions on reinforcement learning for traffic signal control

**DOI:** 10.1371/journal.pone.0277813

**Published:** 2022-11-21

**Authors:** Hyosun Lee, Yohee Han, Youngchan Kim, Yong Hoon Kim

**Affiliations:** 1 Department of Transportation Engineering, University of Seoul, Seoul, Korea; 2 Civil and Environmental Engineering, University of Windsor, Windsor, Canada; University of Shanghai for Science and Technology, CHINA

## Abstract

The increasing traffic demand in urban areas frequently causes traffic congestion, which can be managed only through intelligent traffic signal controls. Although many recent studies have focused on reinforcement learning for traffic signal control (RL-TSC), most have focused on improving performance from an intersection perspective, targeting virtual simulation. The performance indexes from intersection perspectives are averaged by the weighted traffic flow; therefore, if the balance of each movement is not considered, the green time may be overly concentrated on the movements of heavy flow rates. Furthermore, as the ultimate purpose of traffic signal control research is to apply these controls to the real-world intersections, it is necessary to consider the real-world constraints. Hence, this study aims to design RL-TSC considering real-world applicability and confirm the appropriate design of the reward function. The limitations of the detector in the real world and the dual-ring traffic signal system are taken into account in the model design to facilitate real-world application. To design the reward for balancing traffic movements, we define the average delay weighted by traffic volume per lane and entropy of delay in the reward function. Model training is performed at the prototype intersection for ensuring scalability to multiple intersections. The model after prototype pre-training is evaluated by applying it to a network with two intersections without additional training. As a result, the reward function considering the equality of traffic movements shows the best performance. The proposed model reduces the average delay by more than 7.4% and 15.0% compared to the existing real-time adaptive signal control at two intersections, respectively.

## Introduction

In recent years, traffic congestion has been increased by the growing traffic demands. Traffic congestions in urban areas primarily occur at signal intersections, and the operation of the traffic signal control (TSC) is one of factors that cause congestion. Since the traffic network is already socially extended, the existing TSCs have to be used in a more intelligent way to control the level of congestion [1]. Many TSC systems have been studied extensively and have been found to be a very useful tool in a great variety of context. These traditional approaches are classified as fixed-time, actuated, and adaptive controls [2]. The fixed-time controls repeat several pre-defined signal times according to traffic patterns, and they are convenient in places where traffic patterns are constant or small perturbations in a uniform traffic flow. However, it is limited to providing adaptive signals for dynamic traffic conditions. The actuated signal control is used to improve efficiency in the main road by skipping unnecessary signal time of minor road or left-turn. However, they lack of active responding to real-time traffic information. The adaptive signal control system adjusts the signal timing plans based on the current traffic conditions, demand, and system capacity in real time [3]. However, these algorithms are also limited to transfer to different local traffic conditions since it is designed to fit specific traffic conditions. It is usually impractical to cover a variety of traffic conditions.

Recently, due to the evolution of artificial intelligence, there has been increasing attention on solving these issues by combining artificial intelligence technology and TSC. Among these methods, reinforcement learning (RL) is particularly suitable for incorporating into TSC because it can adapt to various traffic conditions and optimize itself for various environments with no prior knowledge. In addition, RL-TSC exhibits online learning capability and continuously improves its performance after the system is deployed by adapting to changing traffic demands [4]. To develop a robust and efficient RL framework for traffic signal control, constructing relationships between signal control actions, their effects on the performance (state), and the estimated potential rewards are important.

In the context of RL-TSC design, there are some practical limitations in real-world applications. First, most studies did not capture each individual traffic movement’s congestion indexes in reward functions design. The reward component affects the final goal of the RL model. Most reward functions utilize delay [5–10], queue length [11–15], and throughput [16, 17]. Although these researchers have focused on improving the performance of the aggregated values at the intersection, research on the congestion indexes for each movement is limited. The existing method of setting a reward function may cause signal control to concentrate on movements with a large traffic volume and number of lanes.

Second, there is a practical limitation on the data collection. The state variables (e.g., waiting time, queue length, etc.) represent the current performance condition in an RL environment and can be collected from a CCTV camera, radar, or inductive loop [6, 8, 9, 18–22]. The queue length is the most used for the state variables [5, 6, 11–13, 17, 23–26]. However, observing the entire link with these sensors are limited due to the line of sight and resolution of sensors. Therefore, it is important to develop a practical way to address the detection limit of the real-world sensors.

To bridge the aforementioned practical gaps in the literature, we propose an RL-TSC model that considers the real-world applicability: i) the detection area of the vehicle and ii) the dual-ring signal system for the equality between the traffic movements. Our models adopt the deep deterministic policy gradient algorithm, which is one of the policy gradient methods of deep RL. It can use a continuous action space unlike the deep Q-network algorithm [8, 13, 24, 25]. It is suitable for calculating traffic signals that needs to respond to dynamic traffic flow changes. We define the intersection delay of the reward function by considering the balance of the delay for traffic movements. The reward function includes the entropy of delay while minimizing the newly defined intersection delay. Further in order to overcome the real-world data collection, we predict the density of the entire link using a traffic environment prediction model based on the amount of traffic detected within the detection area of ​​the link in the traffic simulation.

The contributions of this research include: i) balancing the delays in each traffic movement while minimizing the total delay using entropy of delay as the reward function of the RL-TSC models, ii) designing the state of the RL agent considering the real-world applicability, and iii) extending to different types of intersections, pre-training of the models is performed at a simple prototype intersection. The test is performed at the intersections of Daechi Station and Dogok Station in Seoul, Korea by using microscopic traffic simulator VISSIM.

The remainder of this paper is structured as follows. Section 2 reviews related work and summarizes the contributions of this research. The framework of the RL-TSC-based deep deterministic policy gradient algorithm is explained in Section 3 and the construction of the proposed RL-TSC models in Section 4. Section 5 presents the prototype learning method and training results, while Section 6 evaluates the proposed models using a real-world isolated intersection. Finally, Section 7 presents the conclusions of this research and further directions.

## Related work

The method of setting performance indexes such as state and reward and selecting an RL algorithm that stabilizes the learning process is important to develop a robust and efficient RL-TSC [27]. Most of the earlier research on RL-TSC considered only two phases, major and minor roads, when setting up the action space [13, 17, 24, 28]. To reflect a separate left-turn, few studies have implemented the ring-and-barrier system. Muresan et al. [25] proposed deep RL methods to implement simple two-phase and full ring-and-barrier controllers. The application of the ring-and-barrier design reduced delays by at least 5% and average queue lengths at intersections by 27%. They define the actions as phase switching, which is the agent decides whether to advance to the next phase or keep the current phase at every second. However, when the phase switching is applied to the real-world, it provides a traffic signal by deciding whether to extend the current phase, so it does not take into account the over-saturated traffic movements that will occur in the future under heavy traffic conditions. Even if the same reward indexes are used, the results can vary depending on the purpose of the signal control. In previous studies, performance indexes were set up by minimizing intersection delays [5–10, 29, 30] and queue lengths [11, 12, 15]. By contrast, equality by traffic movements, with no bias towards one traffic movement, is as crucial as reducing the overall delay at intersections in RL-TSC.

El-Tantawy et al. [31] investigated Q-learning and SARSA among RL learning methods. Each model is compared by defining three states, two actions, and four reward functions. In particular, the reward functions include minimizing the intersection delay, maximizing the reduction in the total cumulative delay, minimizing and balancing the queue length, and minimizing stops. Reducing the sum of the squared maximum queues [32–34] is suitable at intersections with balanced demand. However, it did not consider balancing the distribution of queue lengths among the traffic movements. In order to achieve balancing the queue length, indexes that reduces the degree of scattering between the queue lengths of traffic movements should be reflected in the reward function.

Notably, many of the proposed reward definitions above rely on information that is practically difficult to obtain in a real-world [4]. The density is one of variables to be estimated accurately in real world [35]. The condition is analyzed using an image of the link: a snapshot of the current vehicle’s location, velocity, and waiting time pixelating the surface of the link into small cells. Also, existing research mentions the limitation that loop detectors and CCTV camera detectors can only detect existing vehicles [36, 37]. Therefore, it is necessary to figure out how much performance the agent actually shows only with the state variable or reward function defined by the information collected in the limited detection area.

There are three limitations in existing research. First, most of existing studies considered only the through traffic movements or single-ring when designing the action space. Second, they focus on maximizing the performance of the intersection rather than balancing the traffic movements when designing the reward function. Finally, they design state variables and reward functions without considering the gap between the simulation environment and the real-world, such as the possibility of collecting information in actual and the limit of the detection area. In this research, to compensate for these limitations, the following is performed. First, we designed an action space as a dual-ring traffic signal system that reflects the minimum green time for pedestrians. Second, the reward function is defined to minimize and balance the delay by considering the scattering degree of the delay between the traffic movements. Last, in the experiment section, the agent calculated the optimal signal based on the data collected within the detection area of the simulation like the data collection environment of the real-world.

## Learning algorithm

**Fig 1:**
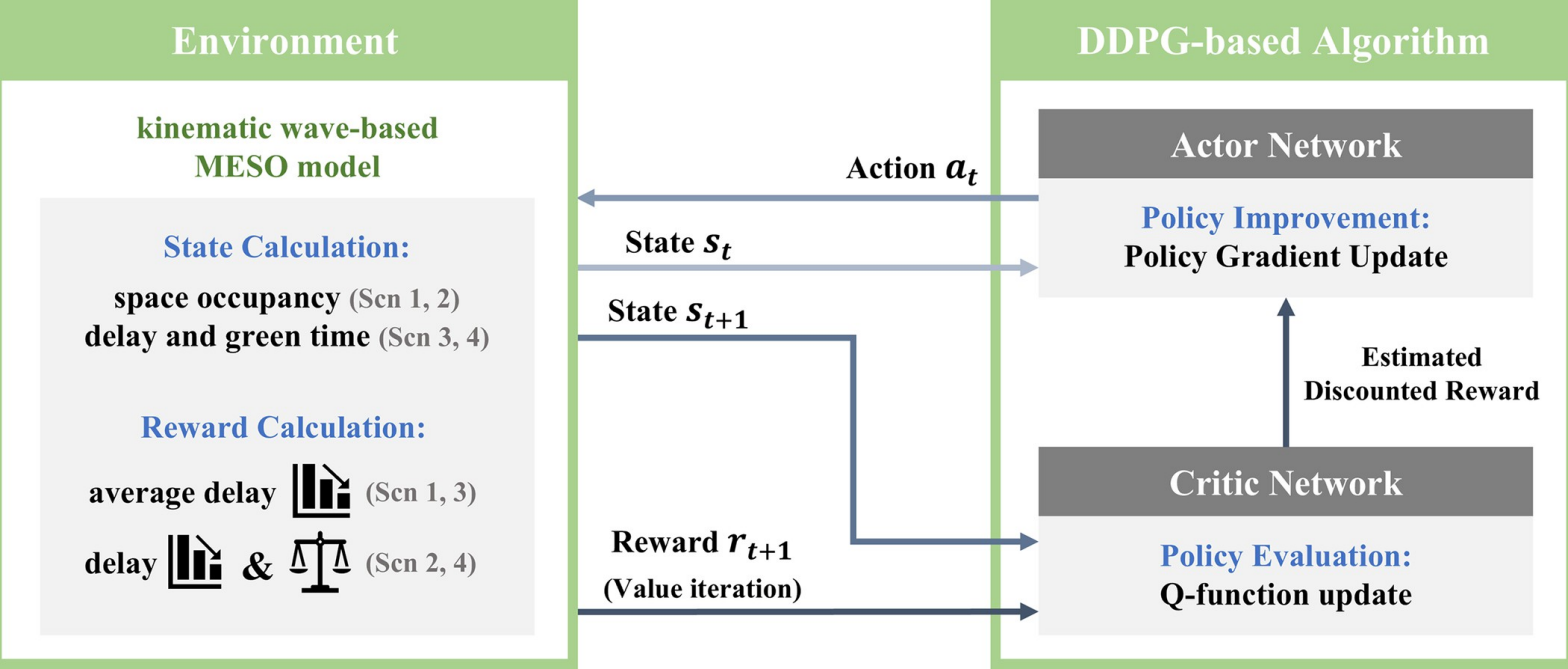
Framework of the proposed signal control algorithm.

We used the deep deterministic policy gradient to learn the agent of the proposed models. It is a model-free, off-policy A2C algorithm using deep function approximators that learn policies in high-dimensional, continuous action spaces [38]. We considered time continuity in TSC and set a continuous action space. The deep deterministic policy gradient algorithm consists of two neural networks: an actor and a critic. Being a policy-based RL algorithm, the agent’s action depends on policy *π*, which maps the state to the action probability distribution. The actor network determines the policy using state *s*_*t*_ as the input and returns the agent’s action *a*_*t*_ to control the traffic signal, as illustrated in Fig 1. The action *a*_*t*_ is selected based on the final output of the actor network and noise *N*_*t*_. The noise makes the agent attempt new explorations, which is a major motivation to learn in continuous action spaces. The exploration policy *μ*′ is then computed as

$$\mu^{\prime }(s_{t})=\mu (s_{t}|\theta_{t}^{\mu })+N_{t}.$$ (1)

The actor network is learned by the policy gradient method, which adjusts the policy parameter *θ* to maximize the objective function *J* using the differential of *J*. The gradient of the expected return for agent ∇_*μ*_*J*(*θ*) can be rewritten as

$$\nabla_{\theta^{\mu }}J\approx \frac{1}{N}\sum_{i}\left[\nabla_{a}Q(s,a|\theta^{Q}){|}_{s=s_{i},a=\mu (s_{i})}\nabla_{\theta_{\mu }}\mu (s|\theta^{\mu }){|}_{s_{i}}\right],$$ (2)

where *N* is the mini batch size.

A new situation *s*_*t*+1_ occurs in the environment due to the action *a*_*t*_ returned from the actor network, and reward *r*_*t*+1_ is given accordingly. Thus, the values of state and action are evaluated as a Q-function *Q*(*s*,*a*) in the critic network. The critic network uses the Bellman equation to learn and outputs the target *Q*-value. Similar to the deep Q-network, the critic network updates the parameters to minimize the loss value of the mean square error. The loss value is given as

$$L(\theta^{Q})=\frac{1}{N}\sum_{i}{(y}_{i}-{Q(s_{i},a_{i}|\theta^{Q}))}^{2},$$ (3)

where *y* is the target value or the expected return computed as

$$y_{i}=r_{i}+\gamma Q^{\prime }(s_{i+1},\mu^{\prime }(s_{i+1}|\theta^{\mu \prime })|\theta^{Q^{\prime }}),$$ (4)

where γ is the discounting factor that reduces the scalar value of future rewards.

The actor network improves policy by approximating the updated Q-network with parameters adjusted in the critic network.

The deep deterministic policy gradient algorithm adopts copied neural networks used to compute the target values to maintain the learning stability. *Q*′(*s*,*a*|*θ*^*Q*′^) and *μ*′(*s*|*θ*^*μ*′^) are copies of the respective networks. It allows the trained network to be tracked slowly, the parameters of networks are updated.

### Agent design

#### Environment prediction model

RL’s environment interface interacts with the agent to help the agent achieve its goals. When the agent selects an action, the environment responds and presents new situations to the agent, and these processes are continuously repeated [27]. Because the environment obtains the rewards that the agent wants to maximize, the prediction of the environment is important in determining the agent’s performance.

In this study, a kinematic wave-based mesoscopic model [39] was used to predict the environment. The mesoscopic model is a delay estimation model that can reflect over-saturated conditions at isolated signalized intersections where the through-flow and left-turn flows are separated. This model is based on the kinematic wave model [40], which is a generalization of the cell-transmission finite difference equation and derives shockwaves as traffic volume and density propagation in units of cells. Further, the model is more sensitive to over-saturation situations than the existing kinematic wave models as it considers FIFO and non-FIFO conditions, and is faster than the micro traffic simulation models. By comparing the model with the microscopic simulation Vissim, Lee et al. [39] demonstrated that the model returns more realistic results than the Highway Capacity Manual (HCM) delay model. In particular, when the volume/capacity rate is 1.65 for left turn and 0.62 for through in a single lane with a left turn bay, the through-flow is also congested as the lane is blocked by left-turning vehicles. However, since the HCM delay model calculates the delay of each movement based on volume/capacity rate without reflecting the FIFO situation, the accuracy of HCM delay model is 76.9% for left turn and 32.5% for through compared with the average delay of Vissim. In contrast, the accuracy of the kinematic wave-based mesoscopic model is 91.5% for left turn and 94.7% for through compared with Vissim. By implementing the over-saturation condition caused by the effects of through and left-turns as a cell transmission method, the kinematic wave-based mesoscopic model shows results similar to the microscopic simulation Vissim. Thus, this model can predict an agent’s environment using geometric structure data, real-time traffic demand, and traffic signal data from isolated intersections.

**Fig 2:**
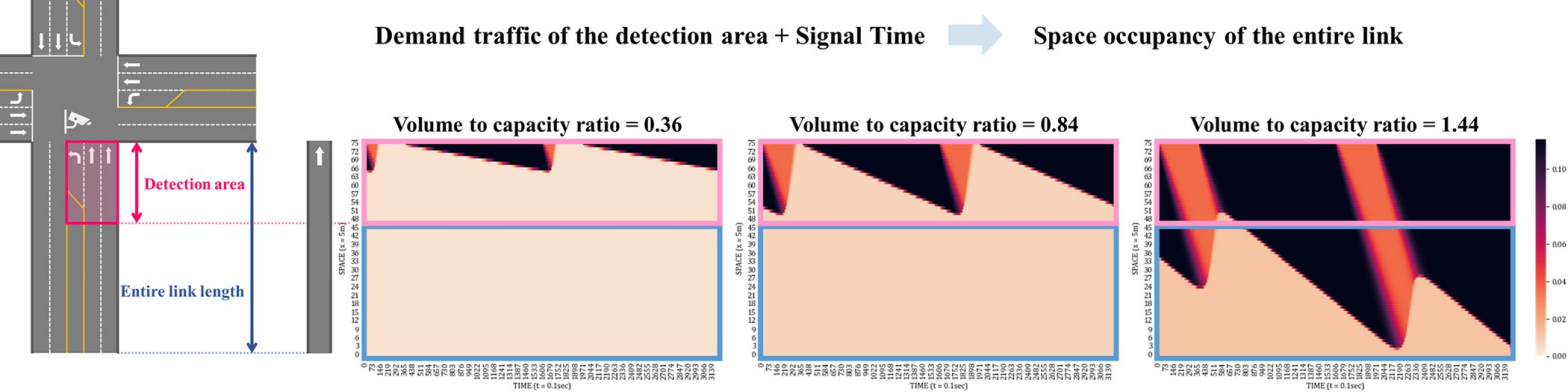
Calculation of the space occupancy based on the collected traffic and signal times.

Many researchers have attempted to estimate space occupancy due to limitations of the traffic detection area in the real world [41]. To overcome this, we obtained the space occupancy of the entire approach link from the kinematic wave-based mesoscopic model using the real-time demand traffic detected in a certain detection area. To verify the estimated space occupancy, the space occupancy based on the calculated area was compared under various traffic conditions: undersaturated traffic condition (volume to capacity is 0.38), near-saturated traffic condition (volume to capacity is 0.84), and oversaturated traffic condition (volume to capacity is 1.44). The space occupancy of the entire link was obtained from the kinematic wave based mesoscopic model with the demand traffic volume collected from a detection area of approximately 150m in the Vissim com interface as shown in Fig 2.

Under all traffic conditions, the space occupancy of 150m, the detection area, was calculated to be larger than the space occupancy of the entire link. This is because the influence of vehicles stopped by the traffic signal is greater when the detection area is limited. The space occupancy of the limited detection area was overestimated by 108.6%, 109.1%, and 33.9% in unsaturated, near-saturated, and oversaturated conditions, respectively, compared to the space occupancy of the entire link. It was confirmed that the error rate of the space occupancy estimated by the mesoscopic model was less than 10% when compared with the space occupancy of the entire link obtained from Vissim. While the space occupancy obtained from only the detection area was overestimated, the space occupancy of the entire link in the mesoscopic model was highly accurate. Therefore, the mesoscopic model enables the agent to learn relatively accurately by efficiently returning the traffic conditions according to the actions taken by the agent.

#### States space

**Fig 3:**
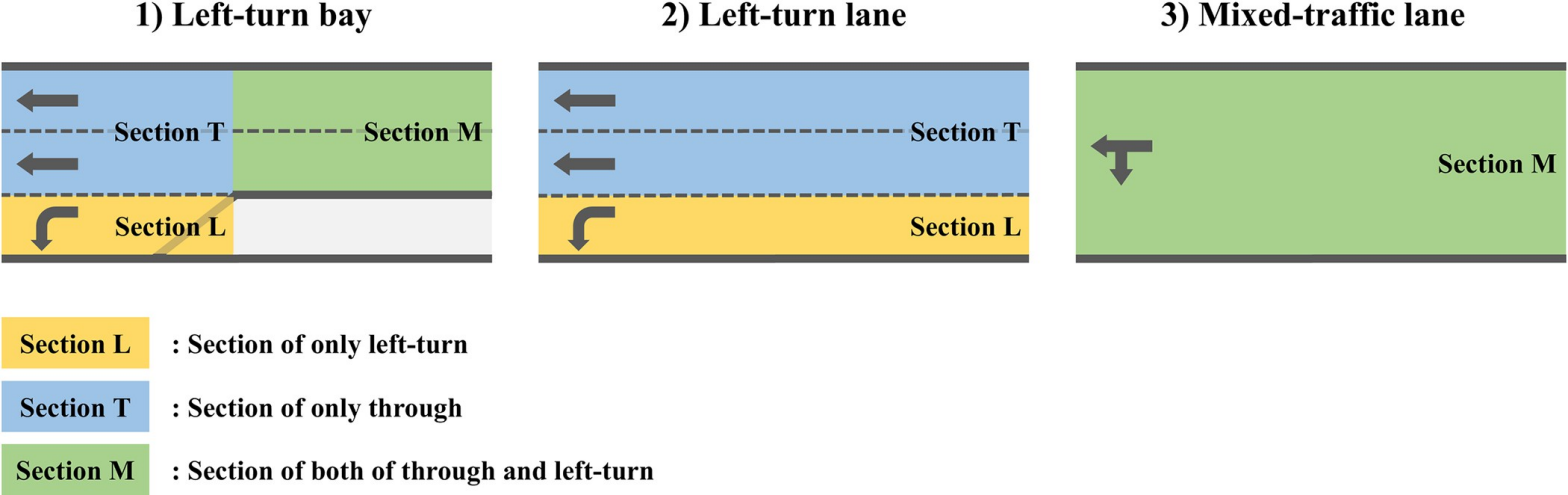
Section division based on lane structure.

We define the state as space occupancy which is a variable representing the traffic conditions of each link intuitively. Space occupancy is calculated using the density of each section obtained from the kinematic wave-based mesoscopic model, which predicts an agent environment. On any approach link *i*, sections can be divided according to the shape of the lane, as shown in Fig 3. The sections include left-turn, through, and mixed sections (both left-turn and through), and the space occupancy is presented as ${occ}_{t}^{i,L}$, ${occ}_{t}^{i,T}$, and ${occ}_{t}^{i,M}$, respectively. In Scenarios 1 and 2, the agent receives 12 space occupancies for the left-turn, through, and mixed sections of each 4-approach link as state s_t_ at each time step *t*,

$$s_{t}=[{occ}_{t}^{1,L},{occ}_{t}^{1,T},{occ}_{t}^{1,M},\ldots ,{occ}_{t}^{4,M}].$$ (5)

#### Actions space

The action definitions of RL-TSC include phase switching, phase selection, and phase duration. Phase switching involves expanding the current phase or skipping unnecessary phases in response to changes in traffic conditions [5, 6, 10, 13, 24, 25, 28]. This method cannot change the order of phase sequences unless the phase is skipped. Phase selection pre-sets executable phase combinations and then maintains the phase similar to the green time of the combinations by selecting phase among candidates [11, 15, 29, 42]. However, phase switching and phase selection may change the cycle time. Moreover, the phase duration determines the length of green time that lasts for each direction [8, 9, 17, 22, 43, 44].

**Fig 4:**
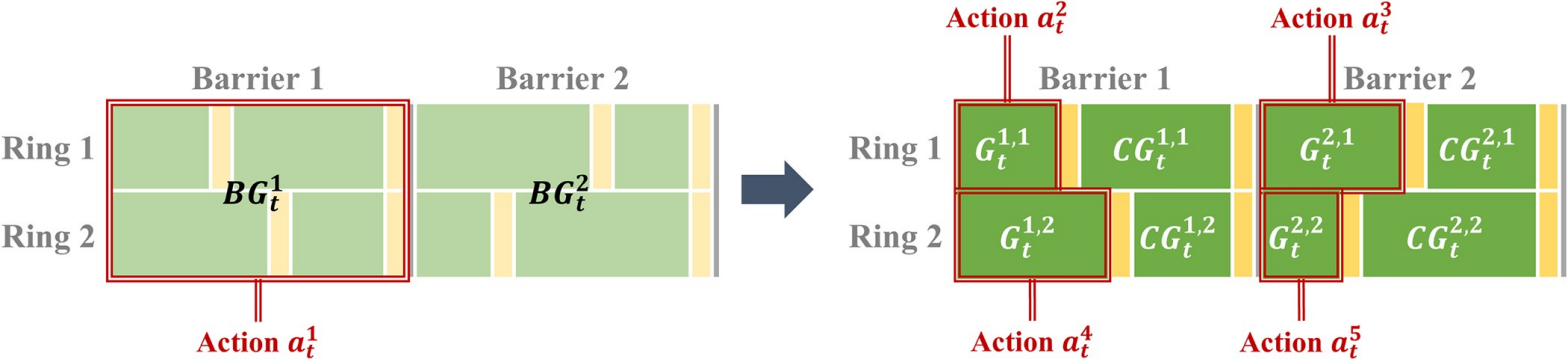
Method of splitting green time according to actions.

We adopted a phase duration that fixes the cycle time to prevent frequent cycle time fluctuations and consider coordination when expanding into the network in the future. When setting up the action space, the dual-ring signal system is considered to reflect the real situation. In addition, the minimum green is set to ensure the pedestrian signal. For the movement with a pedestrian crossing, the crossing is guaranteed by setting the time required for pedestrians to cross the width of the lane as the minimum green. The action determines 8 phases: east bound left-turn (EBL), west bound through (WBT), south bound left-turn (SBL), north bound through (NBT), west bound left-turn (WBL), east bound through (EBT), north bound left-turn (NBL) and south bound through (SBT). First, the green time between the barriers is split and then further split for each ring in the barrier, as shown in Fig 4. In addition, the cycle time duration is fixed and is equal to the sum of the barriers.

Action space at time step *t* consists of five components, $a_{t}=[a_{t}^{1},\;a_{t}^{2},\;a_{t}^{3},\;a_{t}^{4},\;a_{t}^{5}]$: action $a_{t}^{1}$ determines the green time of each barrier, and the other actions $a_{t}^{i}(i=2,\ldots ,5)$ determine the green time of eight phases within each barrier. If the range of actions variation is large, rapidly changing traffic signals can confuse drivers. To avoid this problem, SCOOT, an adaptive traffic signal control system developed in United States in the 1970s, allows changes up to 4 s when performing green split [45]. Therefore, we set the action *a*_*t*_ in such a way that it could change up to 5% of the green time in state *s*_*t*_ to gradually adjust the traffic signal over time. As in Eq (6), action $a_{t}^{1}$ determines the green time of barrier 1 of the next step ${BG}_{t+1}^{1}$ based on barrier 1 of the current time step ${BG}_{t}^{1}$. In sequence, the green time of barrier 2 of the next step ${BG}_{t+1}^{2}$ is calculated using Eq (7).


$${BG}_{t+1}^{1}={BG}_{t}^{1}+{BG}_{t}^{1}∙a_{t}^{1}$$ (6)

$${BG}_{t+1}^{2}=C-{BG}_{t+1}^{1}$$ (7)

After the green split between the barriers is over, the phases of each traffic movement (*b*, *r*) are

$$G_{t+1}^{b,r}={BG}_{t+1}^{b}∙\frac{G_{t}^{b,r}}{{BG}_{t}^{b}}∙(1+a_{t}^{i}),$$ (8)

where *b* and *r* represent the barrier and ring, respectively.

#### Reward function

From the perspective of traffic operations, because traffic signals aim to minimize the delay at an intersection, delay can signify the effectiveness of signal intersections. In this study, we define three reward functions with delay as a variable.

#### Reward definition 1—Minimizing the intersection delay of HCM

The first definition aims to minimize the typical intersection delay, which is most often used as a reward function. The intersection delay defined in HCM [46] is the average delay weighted by the approach flow rate for an intersection. As in Eq (9), the reward is calculated by comparing the typical intersection delay *d*^*I*^.


$$r_{t}=\left\{ \begin{array}{l} 1,\;d_{t-1}^{I}>d_{t}^{I}\\ 0,\;otherwise. \end{array}\right.$$ (9)

#### Reward definition 2—Minimizing the proposed intersection delay

When the objective function for optimizing the traffic signal is set as the typical intersection delay, the green time is concentrated on the traffic movements at intersections with many lanes. Therefore, we define the average delay weighted by the approach flow rate per lane for the intersection as the performance measure, and it is computed as

$$d^{I^{\prime }}=\frac{\sum_{i=1}^{8}\frac{{td}_{i}}{n_{i}}}{\sum_{i=1}^{8}\frac{v_{i}}{n_{i}}},$$ (10)

where *td*_*i*_, *n*_*i*_, and *v*_*i*_ are the total delay, number of lanes, and flow rate of traffic movement *i*, respectively.

The second reward is defined as follows:

$$r_{t}=\left\{ \begin{array}{l} 1,\;d_{t-1}^{I^{\prime }}>d_{t}^{I^{\prime }}\\ 0,\;otherwise. \end{array}\right.$$ (11)

#### Reward definition 3—Minimizing and balancing the proposed intersection delay

The third reward additionally considers the balance of the moving flow in Reward Definition 2. The delay entropy is used as an index to determine the equality of delay for traffic movements. The closer it is to 1, the more uniform is the distribution. The entropy of delay *H*_*t*_ is computed as

$$H_{t}=-\sum_{i=1}^{8}p({td}^{i})\log_{8}p({td}^{i}),$$ (12)

where *p*(*td*^*i*^) is the percentage of the total delay in the current phase *i* to the sum of the total delays at the intersection, which is equal to

$$p({td}^{i})={td}^{i}/\sum_{j=1}^{8}{td}^{j}.$$ (13)

The last reward is defined as:

$$r_{t}=\left\{ \begin{array}{l} 3,\;d_{t-1}^{I^{\prime }}>d_{t}^{I^{\prime }},H_{t-1}<H_{t}\\ 1,\;{(d}_{t-1}^{I^{\prime }}>d_{t}^{I^{\prime }},H_{t-1}>H_{t})\;or\;(d_{t-1}^{I^{\prime }}=d_{t}^{I^{\prime }},H_{t-1}<H_{t})\\ 0,\;otherwise. \end{array}\right.$$ (14)

## Model training

**Fig 5:**
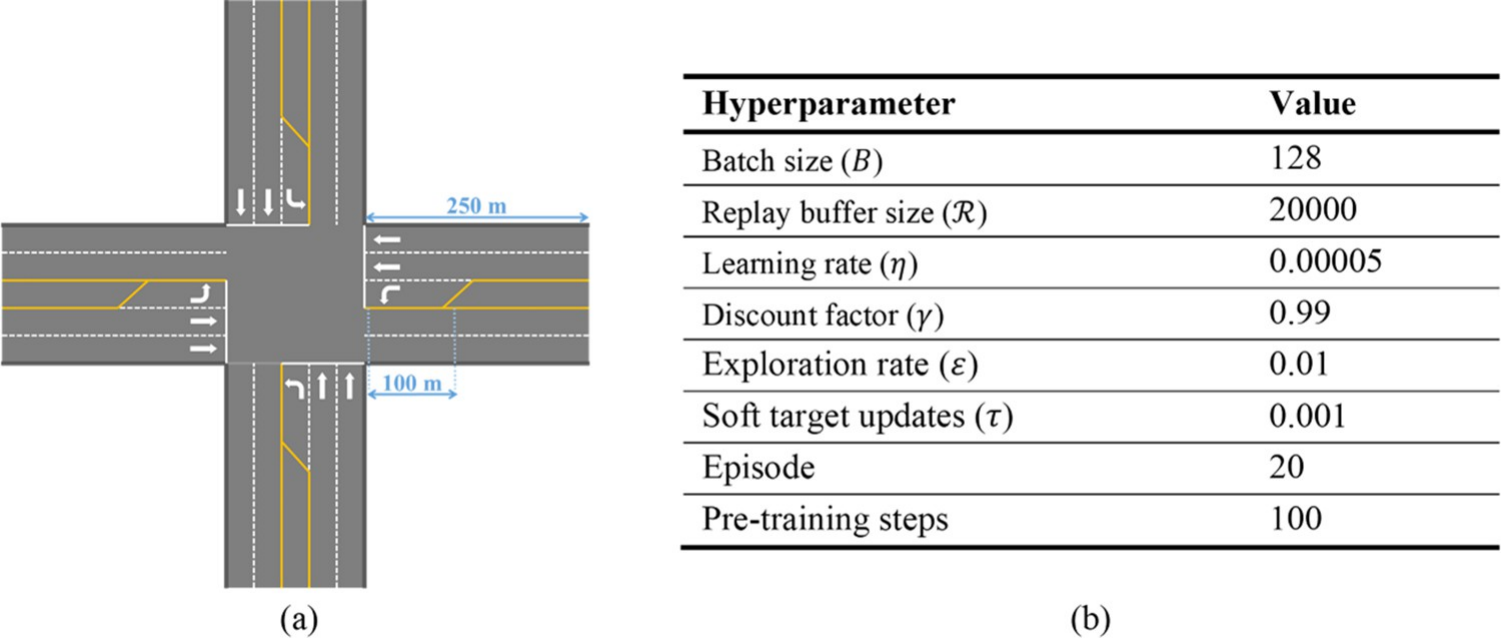
(a) Geometry of the trained prototype intersection; (b) Hyperparameters for pre-training.

The models are trained using Python 3 on Jupyter Notebook. To apply the models to multi-intersection, the models were pre-trained for the prototype intersection where left-turn and through are allowed, as shown in Fig 5(A). By learning various traffic patterns at the prototype intersection, it is possible to apply the q-value of prototype learning without additional learning based on the data per lane even at a four-way intersection with different number of lanes. This method saves the effort of learning the multi-agent from scratch for the corresponding intersections and shortens run time. However, since the action space depends on the traffic signal operation system, it is necessary to implement a separate prototype intersection and carry out pre-training for a single-ring signal system or the three-way intersection. The prototype intersection has eight phases of barrier and dual-ring systems. The cycle time was fixed at 120 s, initial green time for all phases was 27 s, and yellow time was 3 s. The minimum green time was 10 s for the left turn and 20 s for the through to ensure the pedestrian signal. Assuming that the width of each lane is 3.5 m, the total width of the lane that pedestrians must cross is 17.5 m. Assuming a walking speed of 1.0 m/s, a pedestrian signal of at least 19.4 s is required. Since the pedestrian signal operates with the green time of through, the minimum green time for through is set to 20 s. The hyperparameter settings for pre-training are shown in Fig 5(B). The actor network for policy function approximation uses three connected layers with ReLU activations and an output layer with tanh activation for continuous control. In addition, the critic network for *the Q*-value function approximation comprises two connected layers with ReLU activations, in addition to a final dense layer with linear activation.

**Fig 6:**
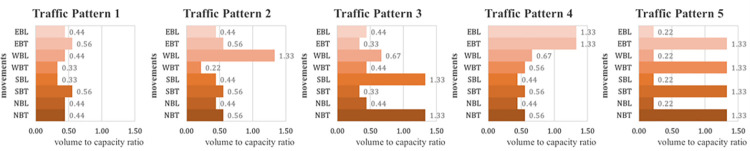
Various traffic patterns for prototype pre-training.

Because the RL agent finds the optimal value, with some uncertainty, it obtains slightly different values each time it performs episodes and steps. Therefore, we repeatedly trained 20 episodes three times to obtain the average value. For multi-intersection extension, the models with three reward definitions are pre-trained using five traffic pattern scenarios with different traffic situations as shown in Fig 6. The figure shows the volume/capacity rate by eight traffic movements for each traffic pattern. Traffic pattern 1 is a low-traffic condition, and Traffic pattern 2 is left-turn over-saturated on the west bound. Traffic pattern 3 and 4 are over-saturated conditions of two traffic movements for each conflict phase (SBL, NBT) and same approach phases (EBL, EBT). Finally, traffic pattern 5 is over-saturated for all through traffic movements.

**Fig 7:**
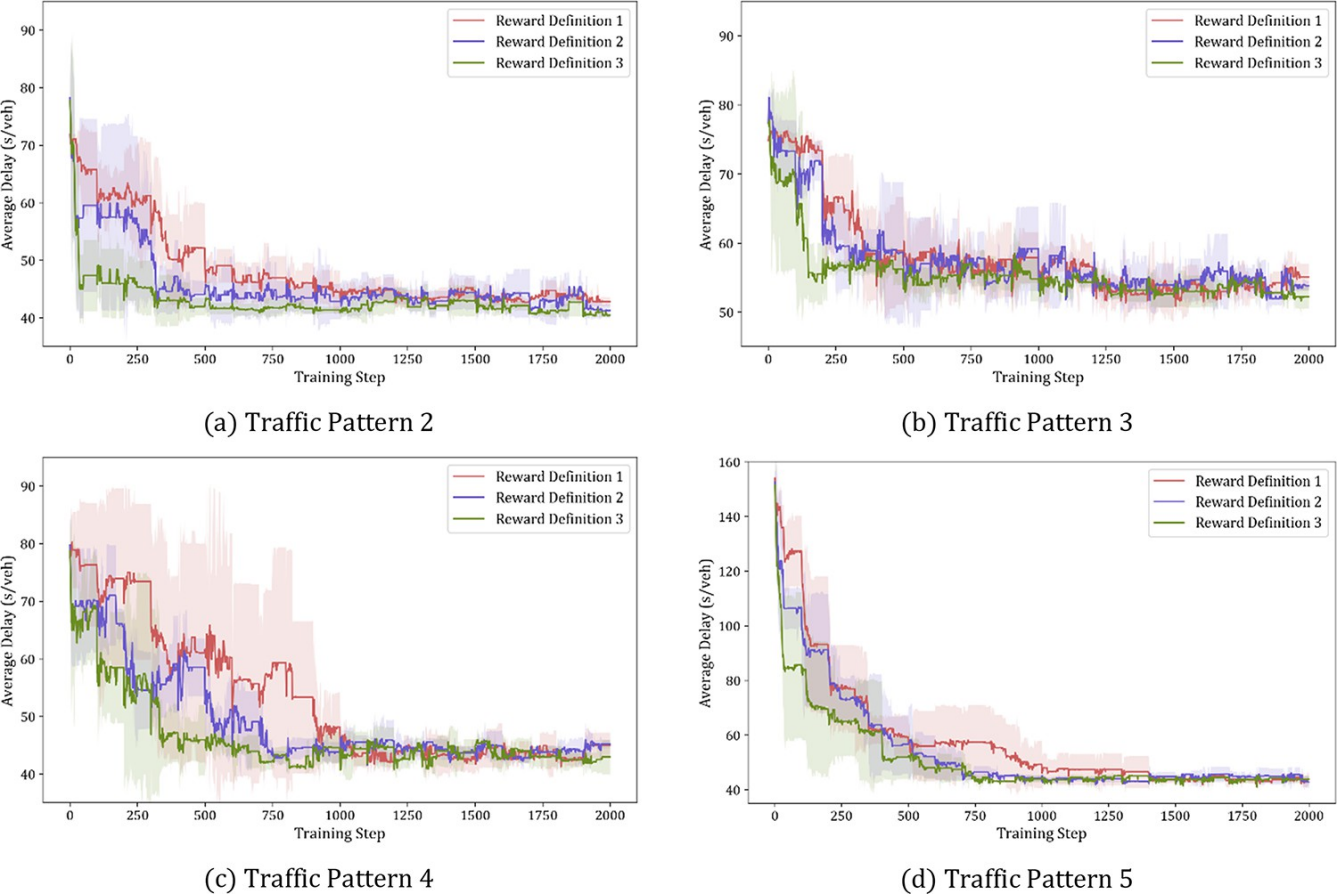
Intersection delay of each traffic pattern for prototype pre-training.

Fig 7 shows the intersection delay in the training results of the model for each reward definition for each traffic pattern. Since the initial delay is small, traffic pattern 1 without significant delay change is excluded from the plot. Traffic pattern 1 with the lowest traffic condition and Traffic pattern 5 with the heaviest traffic condition have a higher rate of convergence than the other traffic patterns. The reason for this is that in Traffic pattern 1 and 5, the traffic volume of traffic movements in the same barrier is uniformly distributed. Traffic pattern 2, 3, and 4 are cases in which traffic is concentrated on one barrier or there is a significant difference in traffic between rings. Although the convergence speed and convergence delay values are different according to each traffic pattern, the convergence speed of the intersection delay is fast in the order of Reward Definition 3, 2, 1. Defining the objective function using the proposed intersection delay enables faster convergence to a lower value than using the intersection delay of HCM. In addition, Reward Definition 3, which set the reward considering the overall delay balance, shows better convergence results in all traffic patterns than Reward Definition 2 that set reward function without delay entropy.

The trained model is verified by implementing a prototype intersection in a Vissim-simulated traffic environment. Traffic pattern 2, 3, and 5 are used as representative conditions for testing. In each pattern, the average reduction rates of the delay of the three reward definitions are 48.2%, 37.4%, and 64.3%, respectively, compared to fixed time control. Although there is a slight difference among the three reward functions, Reward Definition 3 converges to the lowest value in all three traffic patterns. Reward Definition 3 reduces the average delay by 51.5%, 42.0%, and 66.4% in order of the traffic patterns.

## Experiments

### Experimental setup

**Fig 8:**
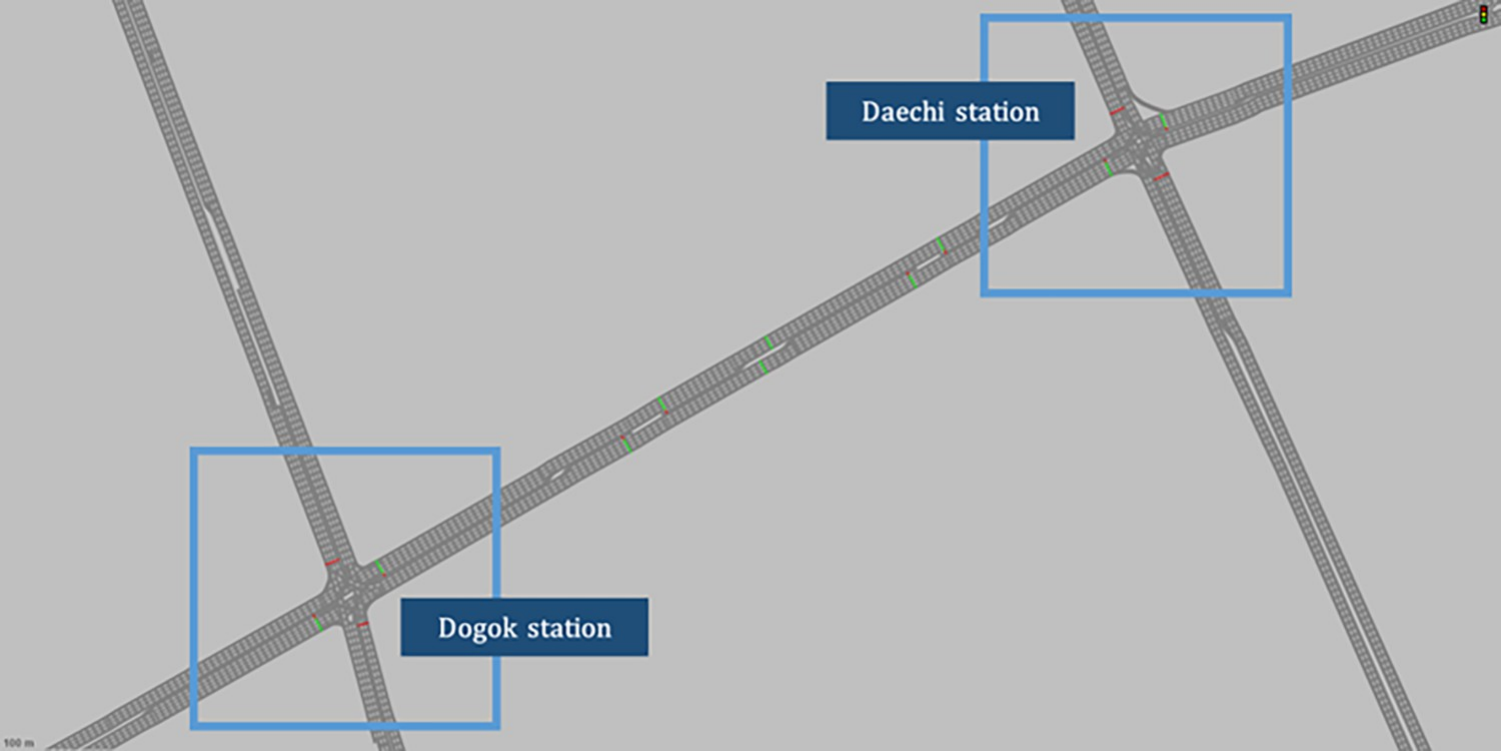
A network with two intersections built in Vissim-simulation.

The proposed TSC based on the deep deterministic policy gradient algorithm was evaluated in a Vissim-simulated traffic environment. The numerical experiments were carried out at Daechi Station and Dogok Station, which are two adjacent signalized intersections located in Gangnam-gu, Seoul, Korea. As shown in Fig 8, there is a crosswalk between the two intersections. The signals of the crosswalks are used as non-optimized fixed signals. The models with Q-values optimized through pre-training are applied to Daechi Station and Dogok Station. These Q-values are self-updated without additional training for each intersection by the self-target update function, a characteristic of the deep deterministic policy gradient algorithm.

Both intersections are dual-ring system with a lag-lag, and the signal time plan from 7:00 AM to 10:30 AM for Tuesday to Friday is presented in Table 1. The real minimum green time was set to 10 s for the left turn and 40 s for through. However, due to the large minimum green, the RL-TSC performance between the reward definitions shows a difference of less than 3%. It is difficult to distinguish whether the reason for not giving as much green time as required for left turn is the difference in reward definition or to ensure the minimum green time for through. To compare the performance of each design scenario, through’s minimum green time was arbitrarily set to 20 s. The hyperparameter settings were the same as in the pre-training stage, as shown in Fig 5(B), but with episodes and steps set to 5 and 40, respectively.

**Table 1:** The fixed time plans of the simulated intersections.

Daechi Station						Dogok Station					
		Barrier 1		Barrier 2				Barrier 1		Barrier 2	
Ring 1	phase	∅6	∅5	∅8	∅7	Ring 1	phase	∅6	∅5	∅8	∅7
		WBT	EBL	NBT	SBL			WBT	EBL	NBT	SBL
	G(Y)	51(4)	26(4)	45(4)	22(4)		G(Y)	50(3)	27(3)	51(4)	18(4)
	Min G	20	10	20	10		Min G	20	10	20	10
Ring 2	phase	∅2	∅1	∅4	∅3	Ring 2	phase	∅2	∅1	∅4	∅3
		EBT	WBL	SBT	NBL			EBT	WBL	SBT	NBL
	G(Y)	59(4)	18(4)	45(4)	22(4)		G(Y)	50(3)	27(3)	40(4)	29(4)
	Min G	20	10	20	10		Min G	20	10	20	10

The experiments were conducted such that the agent derives the optimal signal by receiving the detected traffic volume by Vissim and the previous signal time at each collection cycle as input from the Jupyter notebook. The analysis began after the initial 40 min, from the time for the initial vehicles to be filled, and the analysis time was set to 2 h. The traffic volume detection area in Vissim was set to 150 m considering the actual CCTV-based estimating area. Based on the detected traffic volume, the kinematic wave-based mesoscopic model estimates the environment by deriving spatiotemporal cell density and traffic volume.

### Demand scenario

**Fig 9:**
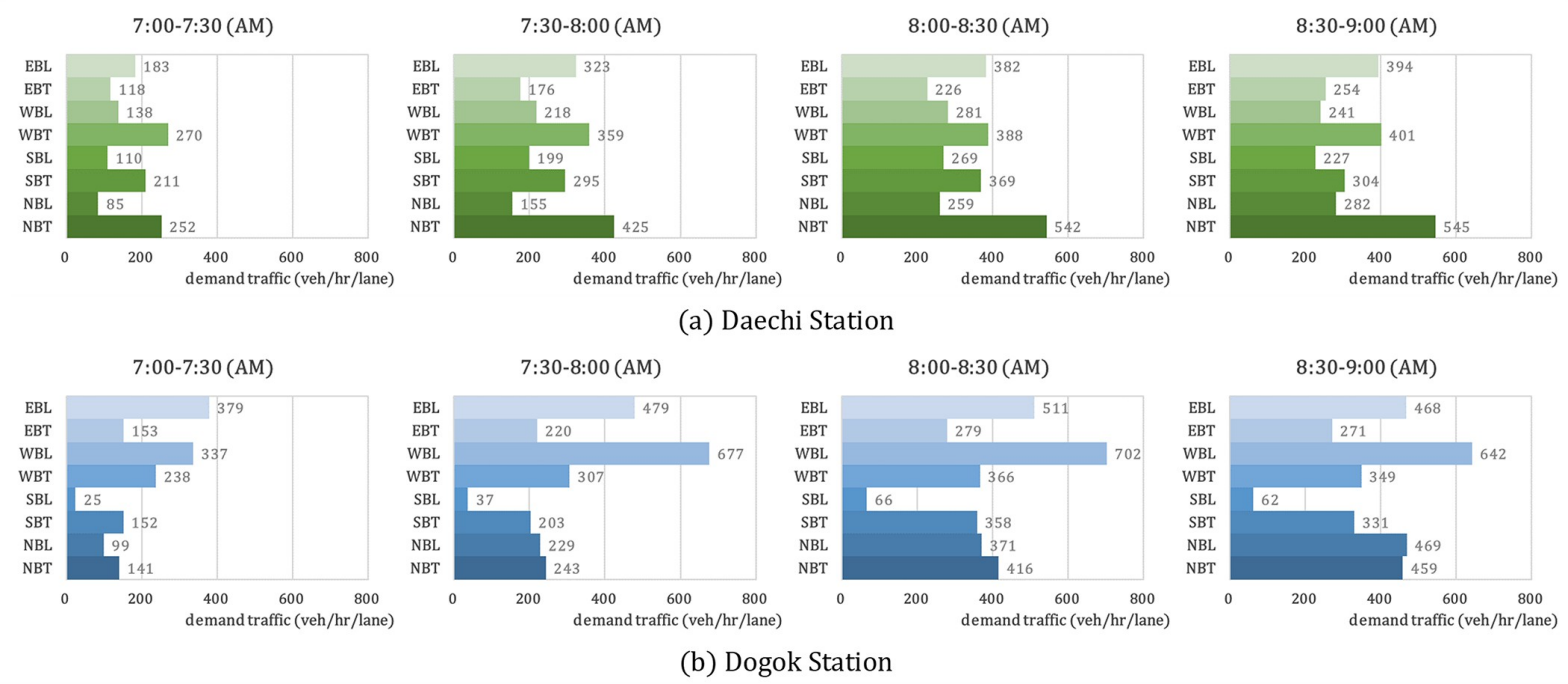
Actual measured traffic patterns of the simulated intersections: (a) Daechi Station and (b) Dogok Station.

We used the traffic volume measured at Daechi Station and Dogok Station from 07:00 AM to 09:00 AM, the peak time on Tuesday morning, as traffic demand data. Traffic demand was aggregated on a 30 min unit and is presented in Fig 9 in terms of traffic volume per lane per hour. The traffic condition increased from 07:00 AM to 09:00 AM. To focus on the performance of RL-TSC, considering only the traffic movements affected by the signal, the right-turn traffic movements were excluded from the simulation analysis.

### Experimental results and discussions

**Fig 10:**
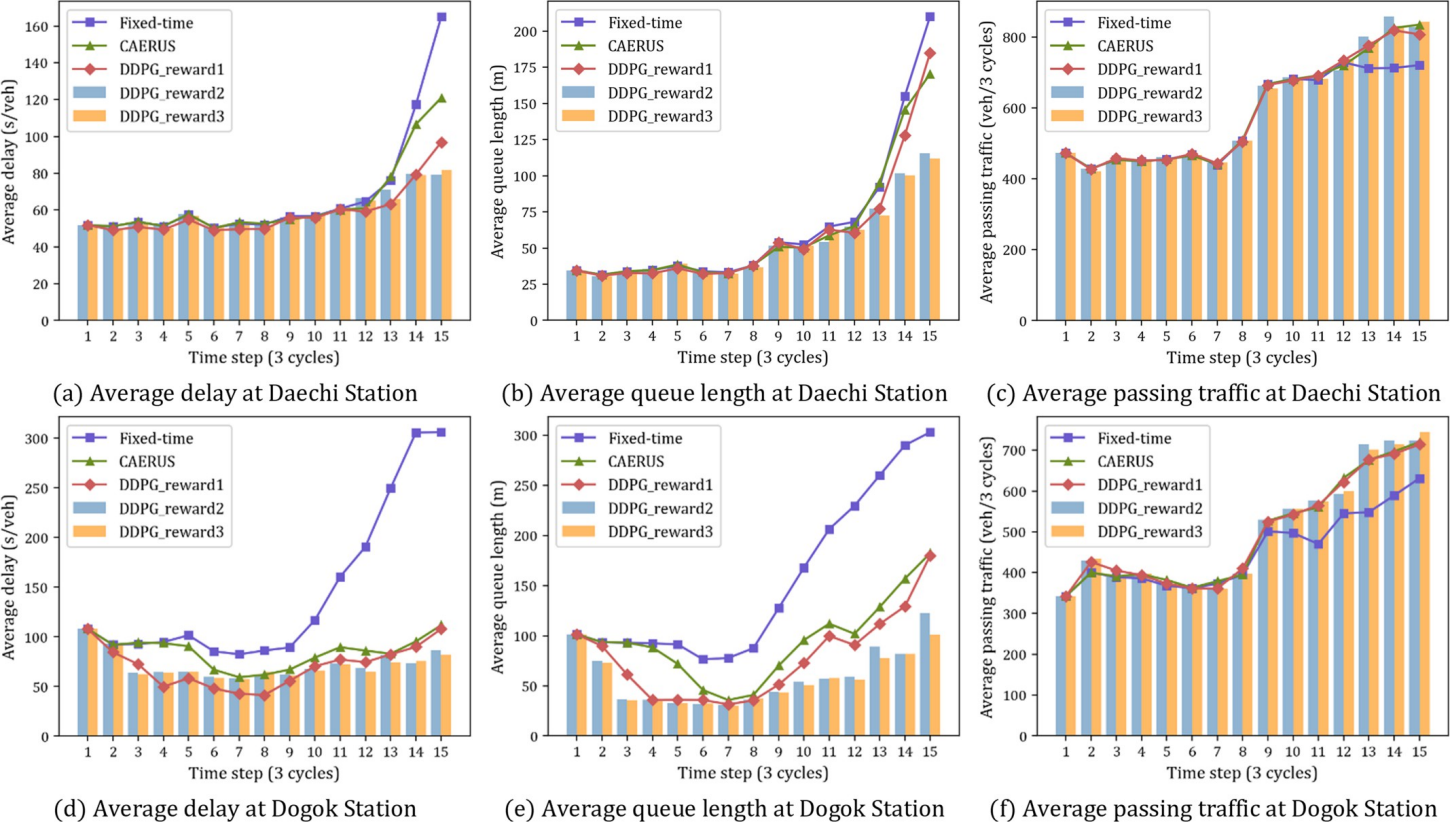
Comparing intersection results for different design scenarios. (a) Average delay at Daechi Station. (b) Average queue length at Daechi Station. (c) Average passing traffic at Daechi Station. (d) Average delay at Dogok Station. (e) Average queue length at Dogok Station. (f) Average passing traffic at Dogok Station.

The proposed models are compared with fixed-time control operated by the signals in Table 1 and CAERUS [47], which is a real-time adaptive signal control that supports volume/capacity rate equalization for each traffic movement. Our models are evaluated as a performance index by applying the optimal signal calculated every three cycles in the Vissim environment. The performance indexes include the average delay, average queue length, average passing traffic as shown in Fig 10.

At both intersections, the traffic volume increases over time, and the heavier the traffic conditions, the better the performance of the proposed models, especially Reward Definition 2 and 3. CAERUS reduces the delay by 5.6% and 40.9% compared to the fixed-time signal at Daechi and Dogok Stations, respectively, while the proposed RL models reduce it by more than 12.6% and 49.7%. The queue length of Daechi Station is reduced by 6.2% in CAERUS and 9.1%, 19.2%, and 19.5% in the proposed models with Reward Definition 1, 2, and 3 compared to fixed-time control. In Dogok Station, the queue length is reduced compared to fixed-time control by 38.3% for CAERUS and 49.4%, 61.3%, and 63.2% for the proposed models with Reward Definition 1, 2, and 3, respectively. For CAERUS and Reward Definition 1, passing vehicles increased by 3.3% at Daechi Station and 9.0% at Dogok Station compared to fixed-time control. For Reward Definition 2 and 3, passing vehicles increased by 3.8% at Daechi Station and 10.2% at Dogok Station.

**Fig 11:**
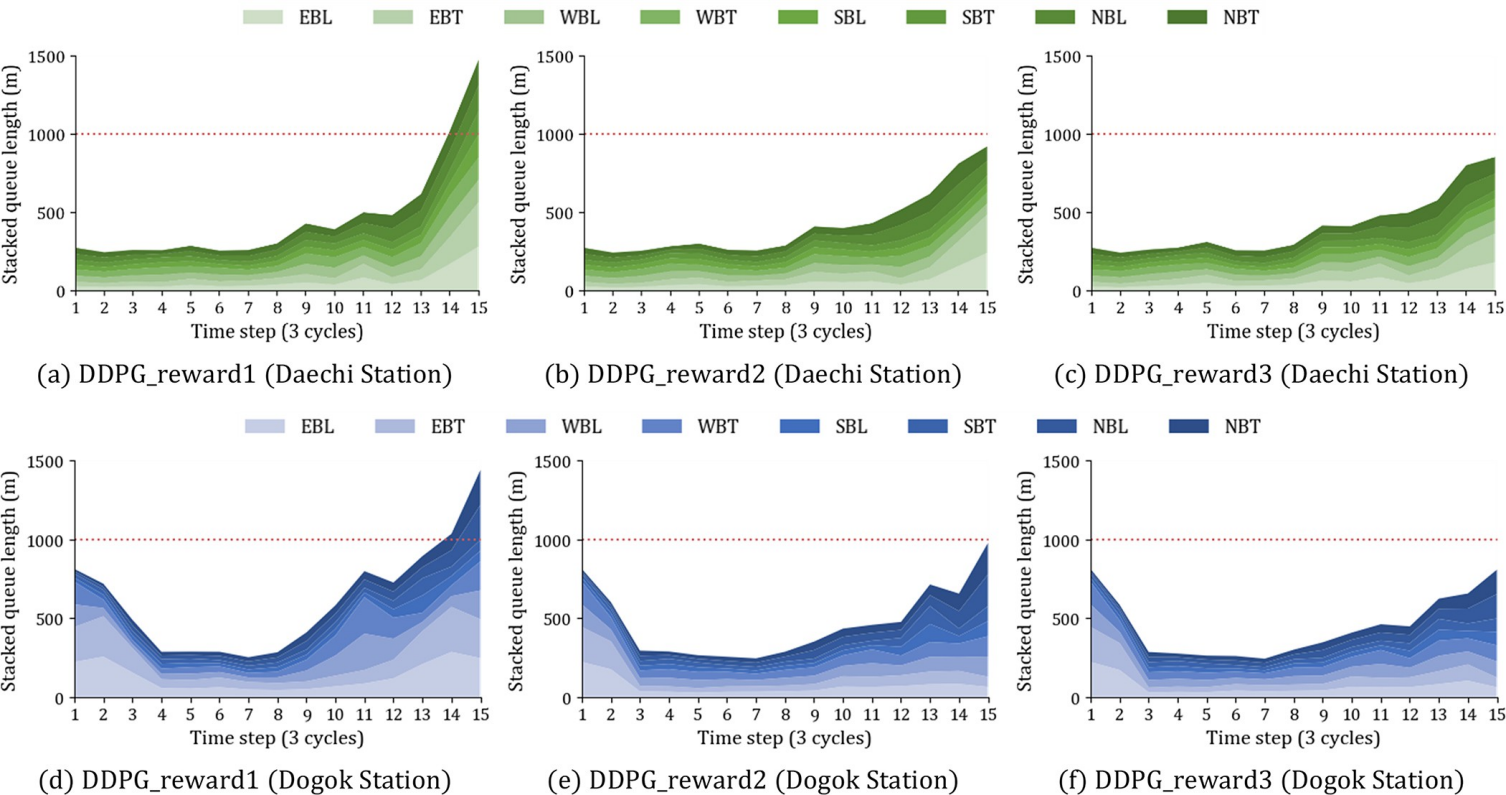
Comparing the stacked queue length of 8 traffic movements for reward definitions. (a) DDGP-reward1 (Daechi Station). (b) DDGP-reward2 (Daechi Station). (c) DDGP-reward3 (Daechi Station). (d) DDGP-reward1 (Dogok Station). (e) DDGP-reward1 (Dogok Station). (f) DDGP-reward1 (Dogok Station).

The intersection delay used in Reward Definition 1 is the average delay weighted by the total traffic flow in all lanes. Therefore, if there are two movements with the same traffic flow per lane, the agent receives a greater reward when giving more green time to the traffic flow with a large number of lanes. Reward Definition 1 performs signal control biased toward specific movements by providing unnecessary green time to movements with excessive traffic on the entire lane. The two models with the best performance, RL-TSC Reward Definitions 2 and 3, show less than approximately 5% difference between aggregated performance indices for the intersection. For evaluating the proposed RL-TSC in detail, the stacked queue length for each traffic movement per time step is analyzed as shown in Fig 11. The difference between the reward definitions is more pronounced in Fig 11 than in Fig 10, which is aggregated in units of the intersection.

As shown in Fig 11(A)–11(C), before the 10^th^ time step, which corresponded to low traffic conditions at Daechi Station, all three reward-defining scenarios show a balanced queue length. However, after the 10^th^ time step the traffic was heavy, and better results are shown in terms of the balance of queue length and total cumulative length in the order of Reward Definition 1, 2, and 3. In the case of Reward Definition 1 at Daechi Station (Fig 11(A)), green time is focused on WBT and NBT with a lot of total traffic, so the queue length of EBL, EBT and NBL with relatively low traffic become more extreme over time. On the other hand, Reward Definition 2 and 3, which use the proposed intersection delay calculated by the traffic per lane as a performance index, show a more equitable and smaller queue length (Fig 11(B) and 11(C)). After the 10^th^ time step of Reward Definition 2, the average of the queue length is 77.3 m and the standard deviation is 30.63 m at Daechi Station. Reward Definition 3 has better performance than Reward Definition 2 with an average of 76.5 m and a standard deviation of 27.5 m. Dogok Station shows a similar pattern to Daechi Station for each reward definition, but with a larger performance difference (Fig 11(D)–11(F)). After the 10^th^ time step, in order of Reward Definition 1, 2, and 3 the average queue lengths are 114.1 m, 77.4 m, and 70.9 m, and the standard deviations are 60.9 m, 22.1 m, and 18.2 m. Reward Definition 3 has the most stable result without fluctuations in values at both intersections, even if the traffic demand changed over the time step.

## Conclusion

In this research, we identified a suitable reward function design for an RL-TSC model. The RL-TSC models are applied directly to various intersections without additional training by using prototype pre-learning for field applicability in the real world. In addition, real-time adaptive signals are evaluated based on the traffic volume collected by the detector on the VISSIM set similarly to the real detection area. The environment is predicted by a kinematic wave-based mesoscopic model, considering the over-saturation and separation of the through-flow and left-turn flow. The three reward definition scenarios are described as follows: (1) minimizing the intersection delay of HCM, (2) minimizing the proposed intersection delay, (3) minimizing and balancing the proposed intersection delay.

For validation, the RL-TSC models are evaluated at the two intersections of Daechi and Dogok Stations with actual demand scenarios. The proposed models trained on a prototype intersection with five traffic patterns showed good performance at two intersections with different initial traffic signals and traffic characteristics without additional training. At Daechi Station, the average delay of all three reward definitions of the proposed model is reduced by 12.6% compared to fixed-time control and 7% compared to CAERUS. At Dogok Station, the average delay is reduced by 49.7% compared to fixed-time control and more than 15% compared to CAERUS. In terms of designing the reward function, the more appropriate objective function is to minimize and balance the intersection delay rather than minimizing the intersection delay. With respect to minimizing delay, we used the average delay weighted by the approach traffic volume per lane as the intersection delay to prevent large concentrations caused by the number of lanes. Moreover, another performance index was set by adopting the entropy of delay to balance the total delay of each traffic movement per lane. In both Reward Definitions 2 and 3, since the reward function is to reduce the intersection delay, the two cases show less than approximately 5% difference in Fig 10, which is aggregated in the intersections. However, the queue length deviation between movements in Reward Definition 3 is smaller than that in Reward Definition 2. This means that although the two reward definitions have similar intersection averages, Reward Definition 2 has heavy congestion in some movements and the green time is concentrated in certain movements. Consequently, this study clearly demonstrated that the scenario considering the equity of delay as the reward provided more stable results for dynamic traffic demand by analyzing the queue length of traffic movements according to each reward definition.

There are some limitations and future research directions. First, in heavy traffic situations, such as the last time step in Fig 11, congestion cannot be solved only by optimizing the green time because the cycle time is fixed. Therefore, future research should consider adjusting the cycle time. In addition, it is essential to set the permission regulations of the overlap phase to prevent driver confusion when applying the proposed RL-TSC models. Furthermore, the proposed RL-TSC models are applied to the lag-lag system in this research and will consider the lead-lead and split system in the future. Ultimately, the proposed RL-TSC models for an isolated intersection should be expanded to include coordination between intersections. Furthermore, when designing Reward Definition 3, we arbitrarily designed rewards as 3, 1, and 0 points to give weight more than Reward Definition 2 when the balancing of movements is satisfied. However, since the speed and degree of convergence of agents varies according to reward assignment, research on parameter optimization for reward assignment is needed in future.
